# Revealing the Viral Community in the Hadal Sediment of the New Britain Trench

**DOI:** 10.3390/genes12070990

**Published:** 2021-06-29

**Authors:** Hui Zhou, Ping Chen, Mengjie Zhang, Jiawang Chen, Jiasong Fang, Xuan Li

**Affiliations:** 1 Key Laboratory of Synthetic Biology, CAS Center for Excellence in Molecular Plant Sciences, Institute of Plant Physiology and Ecology, Chinese Academy of Sciences, Shanghai 200032, China; zhouhui@sippe.ac.cn (H.Z.); pchen@ips.ac.cn (P.C.); zhangmengjie@sippe.ac.cn (M.Z.); 2 University of Chinese Academy of Sciences, Beijing 100049, China; 3 Ocean College, Zhejiang University, Zhoushan 316021, China; 4 Shanghai Engineering Research Center of Hadal Science and Technology, College of Marine Sciences, Shanghai Ocean University, Shanghai 201306, China

**Keywords:** New British Trench, hadal sediment, metagenomics, virus

## Abstract

Marine viruses are widely distributed and influence matter and energy transformation in ecosystems by modulating hosts’ metabolism. The hadal trenches represent the deepest marine habitat on Earth, for which the viral communities and related biogeochemical functions are least explored and poorly understood. Here, using the sediment samples (8720 m below sea level) collected from the New Britain Trench (NBT), we investigated the viral community, diversity, and genetic potentials in the hadal sediment habitat for the first time by deep shotgun metagenomic sequencing. We found the NBT sediment viral community was dominated by Siphoviridae, Myoviridae, Podoviridae, Mimiviridae, and Phycodnaviridae, which belong to the dsDNA viruses. However, the large majority of them remained uncharacterized. We found the hadal sediment virome had some common components by comparing the hadal sediment viruses with those of hadal aquatic habitats and those of bathypelagic and terrestrial habitats. It was also distinctive in community structure and had many novel viral clusters not associated with the other habitual virome included in our analyses. Further phylogenetic analysis on its Caudovirales showed novel diversities, including new clades specially evolved in the hadal sediment habitat. Annotation of the NBT sediment viruses indicated the viruses might influence microbial hydrocarbon biodegradation and carbon and sulfur cycling via metabolic augmentation through auxiliary metabolic genes (AMGs). Our study filled in the knowledge gaps on the virome of the hadal sediment habitats and provided insight into the evolution and the potential metabolic functions of the hadal sediment virome.

## 1. Introduction

The hadal trenches are the deepest part of the ocean, accounting for ~1.5% of the global deep-sea floor and covering ~45% of the oceanic depth range [1]. With unique tectonic, topographic, bathymetric, and hydrographic features, trenches have recently become a hotspot for deep-sea microbial research. Currently, culture-independent high-throughput sequencing and culture-dependent taxonomic characterization have provided insights into the microbial communities and functions of seawater or sediments in hadal environments. Logan et al. compared the microbial community similarities and differences between the Kermadec Trench and the Mariana Trench by amplicon sequencing and culture [2]. Liu et al. characterized the composition and metabolic potential of particle-attached (PA) and free-living (FL) bacterial communities in the Marianas Trench by high-throughput sequencing [3]. Despite evidence that benthic prokaryotes and viruses in the surface centimeters of deep-sea sediments account for 50–80% of the total microbial life on Earth and 10–30% of the total biota [4,5], their diversity and metabolic potential in these extreme environments remain largely unknown. Even less information is available on the role of viruses in this ecosystem and their interactions with their prokaryotic hosts.

Globally, viruses are the most diverse group in the marine ecosystem and are extremely abundant, tallying around 4 × 10^30^ [6], with phages estimated to be 5 to 25 times more abundant than bacteria in the surface layer of the ocean [7]. Viruses influence community turnover and resource availability through a range of interactions with their hosts [8]. Viruses can regulate the number of microbial populations by randomly adsorbing, infecting, and then lysing the dominant host bacteria in the microbial community to proliferate, causing some kind of lesion and death of the host cells. They control the overpopulation of the dominant species and thereby regulate and maintain the structure of the microbial community and the balance of species diversity [9]. During infection, the virus may also affect key steps of cellular processes such as metabolism, division, clustered regularly interspaced short palindromic repeat-CRISPR-associated protein (CRISPR-Cas) immunity, motility, and regulation [10]. In deep-sea sediments, viral infestation reduces prokaryotic microbial heterotrophic products by 80%, releasing approximately 0.37–0.63 gigatons of carbon per year globally, suggesting that viruses play an important role in deep-sea ecosystems [11]. However, little has been reported on the diversity, genetic potential, and biogeochemical impact of viruses in the Hadal Trench sediments at depths greater than 6000 m. Elisabetta et al. examined the distribution, activity, and interactions of viruses and prokaryotes in surface sediments at the bottom of the Japanese, Izu-Ogasawara, and Marianas trenches and nearby abyssal plain sites by physicochemical means [12]. However, limited by methodological means, a veil remains over abyssal virus-host interactions, genetic diversities, and biogeochemical impacts on the hadal environment.

The New Britain Trench (NBT), nearing the land of Papua New Guinea, is located in the northern part of the Solomon Sea [13]. It reaches the deepest depth of ~9140 m at Planet Deep [14]. The special geographical location of NBT makes the trench one of the most frequently seismic places in the world. Compared with the Marianas Trench, the NBT is close to land and can receive more heterogeneous input from both land and ocean. Correspondingly, the waters covered by the New Britain Trench have a higher net primary productivity (about three times higher than the Marianas Trench) [13]. Previous studies have investigated the microbiome within the New Britain Trench waters and sediments [15,16,17,18]. However, no research has been focused on the hadal viral community in the sediment of the New Britain Trench. Particularly, questions about viral components and their metabolic functions in the New Britain Trench sediment remain unanswered. Thus, the hadal viral community in the sediment of the New Britain Trench is still poorly understood. Here, we adopted a deep metagenomics approach to investigate the hadal virome in the sediment of the New Britain Trench (8720 m). We have investigated the main viral components and the versatile metabolic functions of the NBT virome. Extensive comparative analysis with virome in hadal aquatic habitats, bathypelagic and terrestrial habitats were also conducted.

## 2. Methods

### 2.1. Sampling Site Descriptions and Sample Collection

Sediment samples were collected at a depth of 8720 m from the New Britain Trench (154°32.2912′ E, 6°14.4920′ S) in January 2017 during a cruise aboard M/V Zhang Jian. Sediment cores were collected using a box corer (with a base area of 400 cm^2^ and a height of 25 cm) attached to the hadal lander-II, developed by Shanghai Engineering Research Center of Hadal Science and Technology, Shanghai Ocean University. The samples were either frozen at −80 °C before stored at −20 °C or stored at 4 °C for culturing and isolation of microbes.

### 2.2. DNA Extraction, Library Construction, and Sequencing

The column sediments were divided into three layers, the surficial layer N1 (sediment depth 1–5 cm), the mid-layer N2 (5–10 cm), and the deep layer N3 (10–15 cm). According to the standard manufacturer protocol, about 10 g of sediment sample were used to extract DNA for each sediment layer, using the MoBio PowerSoil DNA Isolation kit (MO BIO Laboratories, Carlsbad, CA, USA). Three DNA extractions were performed on every sediment sample and were then mixed for Illumina library construction, which minimizes the variation between different extractions. Two Illumina libraries were built for the extraction mixture of each sediment layer, and two separate runs sequenced each library. The first three Illumina libraries of the three samples were sequenced in one Hiseq run and the second sequence for another run to overcome the variation between different sequencing runs. Notably, PCR amplification was limited to 12 cycles for each Illumina library. The quality and quantity of genomic DNA were examined by an Agilent Bioanalyzer 2100 (Agilent Technologies, Redwood City, CA, USA). Metagenomic shotgun sequencing was performed on the Hiseq Xten instruments (Illumina, San Diego, CA, USA), with 2 × 150 bp paired-end reads.

### 2.3. Sequence Processing and Assembly

Raw sequence data were first processed using Trimmomatic (version 0.38) to remove adapter sequences and low-quality bases (Q20) [19,20]. Putative contaminated sequences, like plasmid sequences, human sequences, etc., were removed with bowtie2 (v2.3.4.1) [21]. Duplicated reads generated by PCR amplification were removed using Fastuniq [22]. The final cleaned reads were assembled using MEGAHIT (v1.1.3) [23] with the following parameters: —k-list 21, 29, 39, 59, 79, 99, 119, 127, 139.

### 2.4. Taxonomic Classification of Reads and Determination of Relative Sequence Abundance

The reference database compiled in a GORG-Tropics format, including archaea, bacteria, microbial eukaryotes, and viruses, was downloaded. Taxonomic classification of the sequencing reads was performed using Kaiju (v1.7.0) [24] with greedy-5 mode against the reference database. Reads were assigned with the taxonomic id and functional annotations based on mapped references. The relative sequence abundance of a phylum, class, order, etc., was assessed by summarizing the total number of the corresponding category assigned reads and dividing it with the total number of assigned reads of all the categories.

### 2.5. Recovering and Annotating Viral Contigs

In order to recover high confidence virus sequences from the assembled contig, we integrated the results of multiple virus software identifications. VirFinder(v1.1) [25], VirSorter (v1.0.5) [26], and VirSorter (v2.1) [27] were employed based on the following criteria: (1) VirFinder score ≥0.9 and *p* < 0.05; (2) VirSorter (v1.0.5) categories 1, 2, 4 and 5; (3) VirSorter (v2.1) score >75; (4) both identified by VirSorter (v1.0.5) categories 1–6 and VirFinder score ≥0.7 and *p* < 0.05; (5) contig length >1 k bp. The final recovered viral contigs were analyzed with the taxonomic classification program Kaiju (v1.7.0) with default parameters [24] to get their taxonomic lineage information.

### 2.6. Comparisons to Viral Sequences from Other Environments and Data Sets

To compare NBT sediment viruses (*n* = 40,267) with other habitats, four ecological environmental virus data included: (1) GOV 2.0 seawater [28] (*n* = 310,218); (2) wet-land sediment [29] (*n* = 3344); (3) Stordalen thawing permafrost [30] (*n* = 1907); (4) cold springs [31] (*n* = 2885). For each viral contig, open reading frames (ORFs) were called using Prodigal v2.6.3 [21] and the predicted protein sequences were used as input for vConTACT2 [32]. We followed the protocol published in protocols.io (https://www.protocols.io/view/applying-vcontact-to-viralsequences-and-visualizi-x5xfq7n (version 5.0, accessed on 1 April 2021)) for the application of vConTACT2 and visualization of the gene-sharing net-work in Cytoscape v3.5.0 [33] (edge-weighted spring-embedded model). Viral RefSeq (v95) was selected as the reference database, and Diamond was used for the protein–protein similarity method. Other parameters were set as default.

### 2.7. Functional Viromics Analysis

For viral function analysis, viral contigs were compared with the eggNOG database (version 5.0, accessed on 26 March 2021) using the eggNOG-mapper (http://eggnog-mapper.embl.de/ (version 2.0, accessed on 26 March 2021)) to do functional annotation. A threshold of 10^−5^ was used to filter the annotation results. Viral ORFs annotated as the CAZymes family genes or categorized into “metabolic pathways” (determined by KEGG kos) in the eggNOG-mapper annotation results were considered as potential auxiliary metabolic genes (AMG) [20,34].

## 3. Results and Discussion

### 3.1. Deep Sequencing of the Hadal Microbiome in the NBT Sediments and Identification of Viral Genome/Metavirome

**Figure 1:**
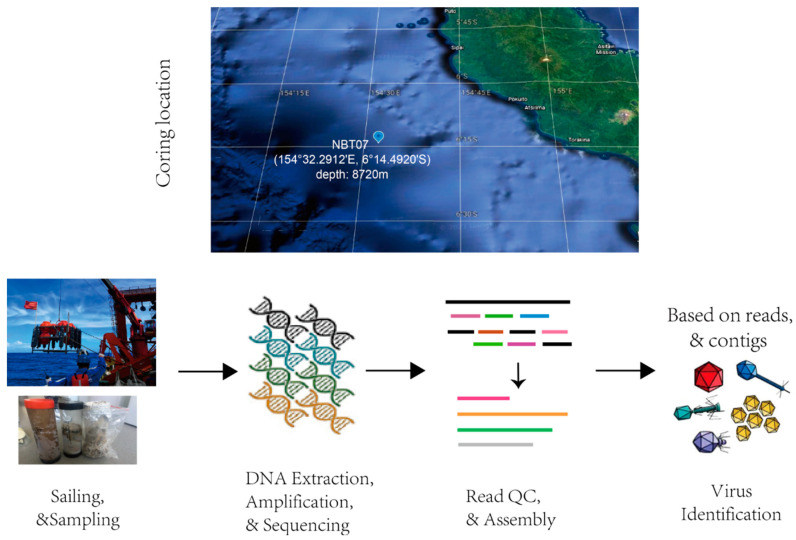
Overview of the study pipeline. Sampling of the hadal sediment in the New Britain Trench (154°32.2912′ E, 6°14.4920′ S) at a depth of 8720 m. One core was collected in January 2017; the coring location is indicated on the top panel.

Sediment samples (depth of 8720 m) were collected from the New Britain Trench in January 2017 (Figure 1, see details in methods) to investigate the community structure, genetic potential, and ecological functions of the New Britain Trench (NBT) sediment virus. Deep shotgun metagenomic sequencing was performed on the NBT sediment samples to study the hadal virome (Figure 1). We divided the sediment samples into three depth layers, i.e., the surficial layer NBT-1 (sediment depth 1–5 cm), the mid-layer NBT-2 (sediment depth 5–10 cm), and the deep layer NBT-3 (sediment depth 10–15 cm). The surface layer of 0–1 cm was re-moved to avoid potential environmental contamination. The meta-genome DNA of each sample layer was then extracted with the proto-cols described in Methods (see section DNA extraction, library construction, and sequencing). Two independent DNA sequencing libraries were built for each sample layer. For each DNA sequencing library, a total of 20~30 Gb sequencing data were obtained. Finally, we obtained ~50 Gb sequencing data for each layer of the NBT sediment (see details in Supplementary Table S1).

Virus identification was based on both the viral reads and the final assembled contigs (Figure 1). After removing adapters and low-quality reads, the sequencing reads were computationally identified and classified by Kaiju [24] based on the least common ancestor (LCA) algorithms. Using the National Center for Biotechnology Information non-redundant (NCBI nr) as the reference database, a total of ~29 M reads were identified and classified as viral sequence fragments. These viral sequences accounted for 0.01% sequence abundance in the NBT sediment microbiome. After co-assembling the total sequencing reads from the samples of the three layers, we integrated three virus contigs classification methods, i.e., VirFinder [25], VirSorter [26], and VirSorter2 [27], to identify the NBT viral contigs (see Methods for details). Then, 714 viral sequences (length >1 kb) were identified by Virsorter, which belonged to categories I, II, IV, and V for “most confident” and “likely” predictions. 13,550 and 29,023 viral sequences (length >1kb) were identified by VirFinder (score ≥0.9 and *p* < 0.05) and Virsorter2 (score > 75), respectively. Finally, 40,267 contigs greater than 1 Kb (Supplementary Table S1) were identified as viral contigs in the NBT sediment sample.

### 3.2. Compositions of the Hadal Viruses in the NBT Sediment Communities

To investigate the viral composition of the NBT sediments, the identified viral reads were further annotated using Kaiju, and the relative abundance was calculated based on the identified viral sequencing reads.

**Figure 2:**
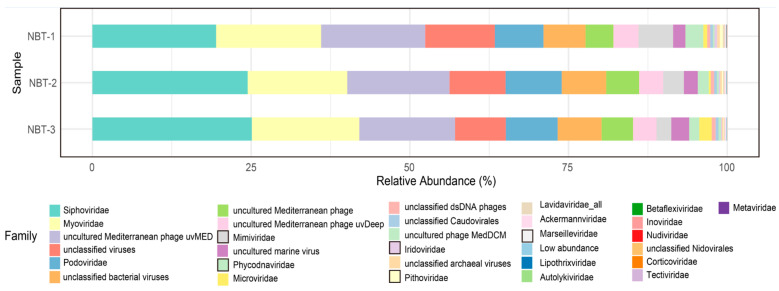
The relative sequence abundance of viral communities of three layers in NBT sediment (top 30). Viral communities shift at the family level. The raw relative abundance data was provided in Supplementary Table S2. Taxonomic assignment of clean reads was performed using Kaiju with NCBI nr database. NBT-1, the surficial layer NBT-1 (sediment depth 1–5 cm); NBT-2, the mid-layer (sediment depth 5–10 cm); NBT-3, the deep layer (sediment depth 10–15 cm).The black frame indicates NCLDV.

A total of thirty major viral families were identified in the NBT sediment (Figure 2). dsDNA viruses, mainly affiliated with the order Caudovirales (naturally infecting bacteria and archaea), were the most abundant. The most dominant viral family in NBT sediment was Siphoviridae, with an average abundance of 42.9% of the total viral sequences, followed by Myoviridae (~16.4% on average) and Podoviridae (~8.2% on average). The abundance of these three families was not significantly varied across the three layers (Figure 2). Notably, we detected a relatively high abundance (~6.10% on average) of the Nucleocytoplasmic Large DNA Viruses (NCLDV) in the NBT sediment, such as Mimiviridae (~3.37% on average) and Phycodnaviridae (~1.98% on average) (Figure 2, color block framed in black). The NCLDV was reported to comprise an expansive group of viruses that infect diverse eukaryotes [35]. The most dominant NCLDV families found in the NBT sediment, Phycodnaviridae, and Mimiviridae, were reported to be eukaryote-infecting viruses [36]. The six main genera of Phycodnaviridae, Chlorovirus (~0.49%), Coccolithovirus (~0.03%), Phaeovirus (~0.01%), Prasinovirus (~0.71%), Prymnesiovirus (~0.01%), and Raphidovirus (~0.05%), were all found in the NBT sediment. Cafeteriavirus and Mimivirus, the two main genera in Mimiviridae, were also found in the NBT sediment and accounted for a relative abundance of ~3.08% and ~0.06%, respectively. Mimiviridae members, such as *Megavirus chilensis* and Tupanviruses, were reported to infect Acanthamoeba in marine [37,38]. Based on our metagenomic analyses, we also detected Acanthamoeba (a relative abundance of ~0.005%) in the NBT sediment, which suggested the existence of eukaryote-viruses interaction in the hadal habitats. The relative abundance of NCLDV was found to have a decreasing trend with increasing depth in the three sediment samples (Supplementary Table S2). Giant viruses tend to be overlooked in previous viral genomic studies since samples are typically filtered according to the preconception of typical virion sizes [39]. Our approach is indifferent to the viral components of different particle sizes. Furthermore, we identified viruses that usually rely on co-infected giant dsDNA viruses of the Mimiviridae for their transmission [40], the Lavidaviridae. The relative abundance of Lavidaviridae accounted for ~0.2% on average in NBT sediment. This virus enhances the survival of the viral host by suppressing giant virus replication [41], which provides new perspectives of virus-host interactions in NBT sediment. Viruses with a relative abundance of less than 1% were also annotated, such as the non-tailed virus Autolykiviridae, the filamentous phages Inoviridae, and small fusiform phage Fuselloviridae (Figure 2 and Supplementary Table S2). Autolykiviridae, a kind of virus with double-stranded DNA (dsDNA), represented a novel family within the ancient lineage of double jelly roll (DJR) capsid viruses. They were reported to have a broad host range in contrast to tailed viruses that kill on average only two hosts in one species [42]. Inoviridae, a kind of single-stranded DNA virus previously reported to be abundant in the oceans [43], were also found in our hadal sediment. Our study comprehensively characterized the compositions of the hadal viruses in the NBT sediment, which opens a new front in the research of microbes-viruses interaction in the hadal habitats. However, the metagenomic strategy can only capture the DNA sequence of the double-stranded DNA (dsDNA) virus and ssRNA (+) virus with dsDNA form during replication. Thus, our study mainly focused on DNA virus research. To further look into the RNA viruses, RNA of the NBT sediment samples should be sequenced and analyzed in the future.

### 3.3. Comparative Analysis of Viromic Compositions between the NBT Sediment and Hadal Aquatic Habitats

To further understand the viromic compositions in hadal sediment, we compared the viromic compositions in the NBT sediment with compositions in hadal aquatic habitats. Due to the lack of metagenomic or viromic data for the NBT aquatic samples, we compared the viromic compositions in the NBT sediment with nine other hadal aquatic samples. These samples included particle-attached (PA), free-living (FL) samples at different water depths (i.e., 4000 m, 9600 m, 10,400 m, and 10,500 m.) in the Mariana Trench [3], and seawater samples at different water depths (i.e., 5000 m, 5700 m, 6000 m) in the Yap Trench [44] (Supplementary Table S3). The relative abundance of virome was calculated with Kaiju using NCBI nr as the reference database (Methods).

**Figure 3:**
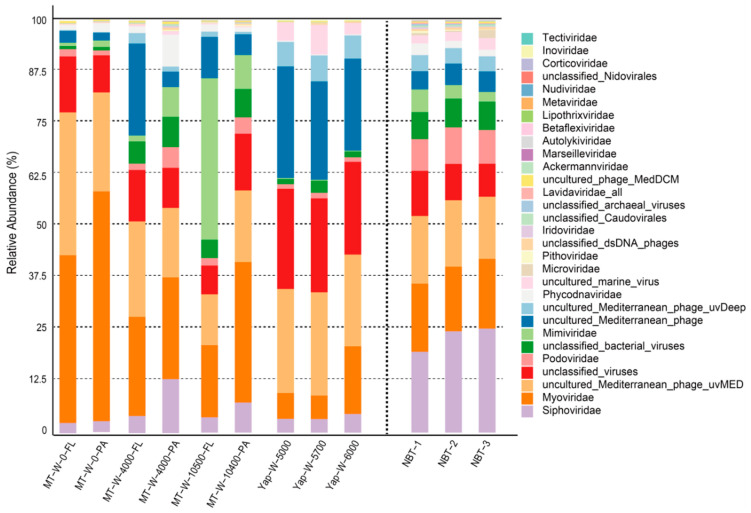
Comparative analysis of viromic compositions between the NBT sediment and hadal aquatic habitats. The relative abundance of the virome was calculated using Kaiju based on the NCBI nr database. Statistical analysis was performed on the relative abundance of the different viruses within the aquatic and sedimental samples (see details in Supplementary Table S3). Sample names are defined by sample location, sample type (seawater is subdivided according to size fraction and sediment is subdivided according to depth if provided), and sampling depth. MT, Mariana Trench; Yap, Yap Trench; W, seawater; PA, particle-attached; FL, free-living. e.g., MT-W-10500-FL is the free-living fraction of Mariana Trench seawater at 10,500 m, NBT-1, the surficial layer NBT-1 (sediment depth 1–5 cm); NBT-2, the mid-layer (sediment depth 5–10 cm); NBT-3, the deep layer (sediment depth 10–15 cm).

Based on the identified viral reads from the twelve hadal metagenomic data sets, variations of virial community structures were observed among the hadal seawater and the NBT sediments (Figure 3 and Supplementary Table S3). Siphoviridae, which accounted for ~20% on average of the three NBT sediment layers, was the most predominant group in the NBT hadal sediments. However, it only constituted up to ~5% on average and ranked sixth of the major viral groups in the hadal seawater (Figure 3 and Supplementary Table S3). Its relative abundance in the hadal seawater was significantly lower than in the NBT hadal sediments (Mann–Whitney Rank Sum Test, *p* < 0.05, Supplementary Table S3). The second and third major virial groups in NBT sediment, i.e., Myoviridae and Uncultured_Mediterranean_phage_uvMED, account for about 16% and 15% of the total virome, respectively, while the relative abundances of these two virial groups increased to higher than 20% in hadal seawater samples (Figure 3 and Supplementary Table S3). Other major virial groups, such as Podoviridae, was constituted up to ~2% on average of the total microbial community in the hadal seawater. In comparison, in the NBT hadal sediments, it accounts for 8% in average relative abundance. The relative abundance of Podoviridae in the NBT hadal sediments is significantly higher than in hadal seawater samples (Figure 3 and Supplementary Table S3). Thus, distinct viral community structures were observed between the hadal seawater and the NBT sediments. The non-metric multidimensional scaling (NMDS) plot can show variations in the microbial community structures of different samples. To further explore the variations of virial community structures among the hadal seawater and sediments, we plot the NMDS plot based on the relative abundance of the virome within each sample (Supplementary Figure S1). The results showed that the three NBT samples are clustering together, and there is a clear separation by NBT hadal sediment samples and hadal seawater samples. Distinct viral community structures were observed between the NBT sediment and hadal aquatic habitats. Previous studies reported that the microbial community structures between the hadal seawater and hadal sediments were distinct [45]. Thus, the hosts of hadal seawater and sediments virome were different, resulting in the distinctive viromic compositions between the NBT sediment and hadal aquatic habitats.

### 3.4. Comparative Analysis of Viromic Compositions between the NBT Sediment and Bathypelagic and Terrestrial Habitats

**Figure 4:**
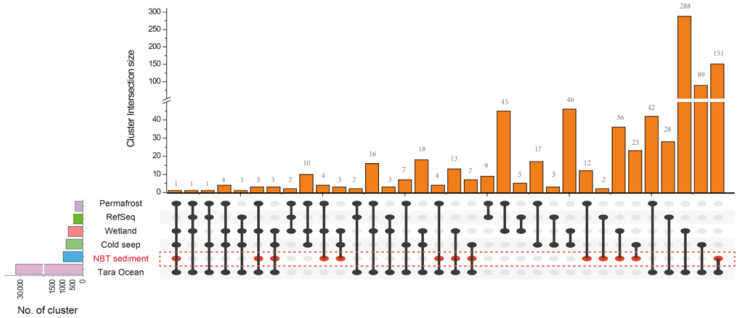
Comparisons to viral sequences from other habitats and data sets. The upset plot shows the comparison of shared viral clusters among four environmental virus data sets (i.e., Tara Ocean seawater, wetland sediment, Stordalen thawing permafrost, Cold seep), Prokaryotic Viral RefSeq, and NBT sediment. Bar size represents the number of viral clusters common to the six sets intersection highlighted using circles below.

Gene-sharing network analysis by vContact2 [32] was performed to have a broad perspective of the hadal sediment virome compared to the virome of the bathypelagic and terrestrial habitats. In the gene-sharing network constructed by vContact2, viruses sharing a high number of genes localize into viral clusters (VCs) could be approximated as belonging to the same genus level [32]. A total of six datasets were analyzed in our study: (i) virus sequences from the global ocean virome (i.e., Tara Ocean dataset, *n* = 310,218) [28]; (ii) virus sequences from cold springs (*n* = 2885) [27]; (iii) virus sequences from wetland sediments (*n* = 3344) [29]; (iv) virus sequences from permafrost environments (*n* = 1907) [30]; (v) NCBI Prokaryotic Viral RefSeq v94 [46]; (vi) virus sequences from NBT sediment (*n* = 40,267). Finally, 33,609 VCs were identified across the six datasets (Figure 4, Supplementary Table S4).

The Tara Ocean dataset was found to have the largest clusters (>30,000 VCs), which showed the highest diversity. This may have resulted from the Tara Ocean dataset from global, long-period, and abundant sampling locations [28]. Only one cluster was found to be shared across all ecological habitats (Figure 4, Supplementary Table S4), which may reflect the high variation of habitats for specific viruses. A total of 1025 VCs were identified in the NBT sediment viruses, of which 756 VCs (73.77%) were unique (Figure 4, Supplementary Figure S2), suggesting that the hadal habitats have evolved specific virus groups that are pertaining to hadal microbial hosts. By comparing the number of VCs shared by NBT sediments and the four bathypelagic and terrestrial habitats, we found that the Tara Ocean had the most shared VCs (*n* = 151), followed by Wetland (*n* = 36), Cold seep (*n* = 23), and permafrost (*n* = 12) (Figure 4). Such a variation in shared VCs may result from the ecological variations between the hadal sediment and other habitats. Only two VCs were found to be shared between NBT sediment and known prokaryotic, viral genomes (RefSeq v94) (Figure 4), indicating a vast majority of the NBT sediment virome were not well classified and characterized. Such an indication was further confirmed by the taxonomic assignment results of the NBT sediment virome by kaiju [24]. Using the least common ancestor (LCA) algorithms in Kaiju, and the NCBI-nr as the viral reference database, only 1.4% of the NBT sediment virome contigs could be assigned taxonomic affiliations. Thus, our findings revealed a lot of previously unknown genomic and taxonomic diversity in the viral communities of the NBT sediment.

### 3.5. Phylogenetic Diversity of the Hadal Virome in the NBT Sediment

Phylogenetic analyses were performed on the major viral groups by using marker genes to assess the diversity and genetic distance among the viruses in the NBT sediment. dsDNA viruses, which are most affiliated with Caudovirales, e.g., Siphoviridae, Myoviridae, and Podoviridae, accounted for the largest fraction in NBT sediment (Figure 2). A previous study reported that the gene coding for terminase large subunits (TerL) could be used to assess the phylogenetic diversity of Caudovirales [47]. Thus, in this study, we used the marker gene TerL to analyze the phylogenetic diversity of Caudovirales in the NBT sediment. In addition, NBT virome contigs homologous to the marker gene TerL (>50% similarity and object coverage ≥ 50) were selected for phylogenetic analysis. Finally, 87 unique NBT sediment viral contigs were used in phylogenetic analyses.

**Figure 5:**
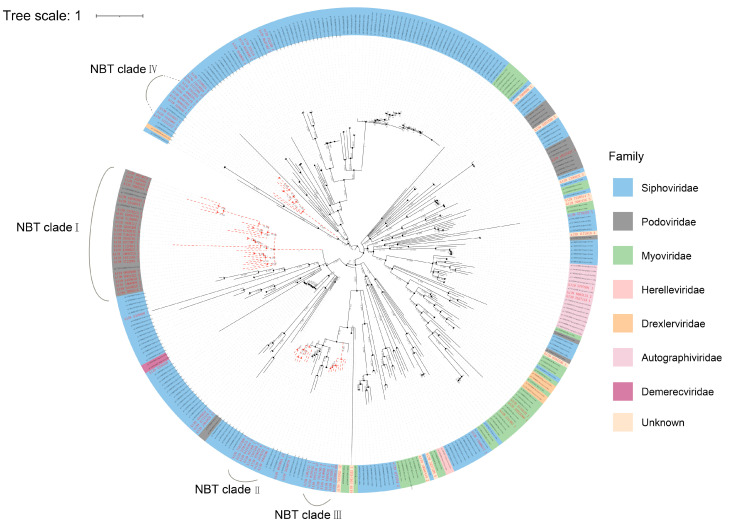
Phylogenetic analysis of Caudovirales based on TerL using the maximum likelihood algorithm. A FastTree approximate maximum-likelihood phylogenetic tree was built using TerL. Reference viral sequences from NCBI are colored in black. Scale bar, one amino acid substitution per site.

As shown in Figure 5, the NBT viral sequences were distributed widely throughout the phylogenetic tree, with most of the sequences affiliated to the Siphoviridae and Podoviridae. Caudovirales are reported to be the extensively studied virus group to date [47]. In this study, most NBT Caudovirales were phylogenetically distant to the known Caudovirales reference sequences and formed four major NBT clades within the Siphoviridae and Podoviridae. This result indicated that there might be an important uncharacterized diversity for Caudovirales in the hadal habitat, such as the NBT sediment in our study. In addition, some viral reference sequences were found to be included in the NBT clades (Figure 5), which provided clues to determine the hosts of phages from these NBT clades. For example, the largest NBT clade, named NBT clade I, included four viral reference sequences whose host was reported to be Betaproteobacteria or Gammaproteobacteria [48,49,50], indicating that members of this NBT clade may mainly infect Proteobacteria.

### 3.6. Functional Analysis of the Hadal Viromics in NBT Sediments

**Figure 6:**
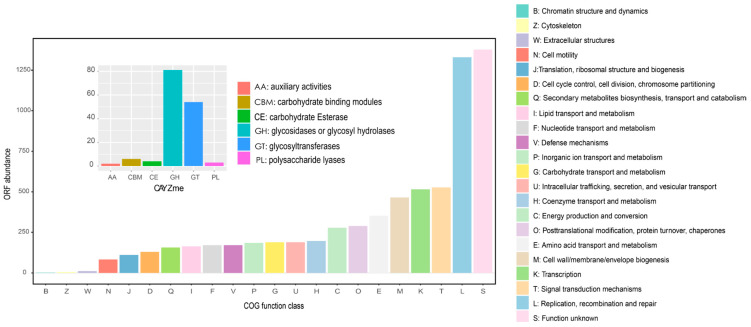
Annotation of viral genomes. COG annotation was performed using eggNOG-mapper. AMGs related to carbohydrate metabolism are illustrated in the inner panel.

For the investigation of gene contents and functions of the NBT sediment viruses, we predicted the genes of the NBT virus contigs and annotated them with eggNOG-mapper using the Clusters of Orthologous Groups (COGs) database [51]. While a large proportion of the predicted genes had unknown functions, high-abundance genes were found in the COG categories of ‘replication, recombination and repair’, ‘signal transduction mechanism’, ‘transcription’, ‘cell wall/membrane/envelope biogenesis’, etc. (Figure 6 and Supplementary Table S5).

Besides the genes for basic viral functions, a special group of virus-encoded genes, the so-called auxiliary metabolic genes (AMGs), can modulate the activities of the hosts upon infection. Overall, the New British Trench sediment viruses tend to encode AMGs for carbohydrate metabolism and cofactor/vitamin, and a significant portion also encoded AMGs for glycan and amino acid metabolism (Supplementary Table S6). The identified carbohydrate metabolism-related genes included 81 GHs (glycosidases or glycosyl hydrolases), 54 GTs (glycosyltransferases), six CBM (carbohydrate-binding modules), four CE (Carbohydrate esterases), three PLs (polysaccharide lyases), and two AA (auxiliary activities). In addition, AMGs related to C1 metabolism (e.g., transketolase), central carbon (e.g., UDP-glucose 4-epimerase), and ABC transporters for carbohydrates were also identified (Supplementary Table S3). Associated hadal sediment microorganisms were reported to be involved in various carbohydrate degradation reactions for processing detrital organic matter supplied from the overlying water column [3,44]. Infection by viruses containing genes related to carbohydrate degradation is consistent, in general, with their supportive role in host metabolism during infection [27,30]. Besides carbohydrate metabolism-related AMGs, we also found AMGs for sulfur metabolism. The *cysC* gene, encoding adenylylsulfate kinase, which reduces *APS* to produce 3′-phosphoadenosine-5′-phosphosulfate (*PAPS*), the second step of assimilatory sulfate reduction, was found in the New British Trench sediment viruses, suggesting possible roles to enhance host sulfate reduction. In this study, we explored the metabolic potential of the hadal virus based on the metagenomic data. However, some shortcomings are still inevitable based on such a strategy. For example, the assembling data might include the prophage data, which would introduce false-positive AMG predictions. Since viruses were difficult to isolate from the hadal sediment, analysis based on metagenomic data is currently the most effective method in exploring the virome in hadal sediment. The metagenomic analyses complement the traditional virology and newer culturomic approaches. However, to further confirm the potential metabolic prediction of the hadal virus based on metagenomic analyses, it would be beneficial to culture these viruses with their corresponding hosts.

## 4. Conclusions

Due to the challenges of sampling and culturing of hadal sediments viruses, the role of viruses in influencing microbial mortality, ecology, and evolution in hadal sediments remains largely uncharacterized to date. This study adopted a deep shotgun metagenomic sequencing strategy to reveal novel, abundant, and diverse viruses of NBT sediments. Double-stranded DNA viruses, particularly those affiliated to the order Caudovirales, were found to be dominated in the hadal viral community. NCLDV that were often missed or underestimated in early studies due to the technique used to capture viral particles via filtering were detected in the NBT sediment in our study. By comparing the hadal sediment viruses with those of hadal aquatic habitats, we found the hadal sediment virome had some common components but was distinctive in community structure. Comparisons with the virome in bathypelagic and terrestrial habitats revealed that the NBT sediment virus community had many novel viral clusters not associated with the other habitual virome included in our analyses. Based on the phylogenetic analysis on the Caudovirales in the NBT sediment, novel diversities, including new clades specially evolved in the NBT sediment habitat, were found. Virus-encoded AMGs, including genes related to carbon and sulfur metabolism, may enhance metabolism in prokaryotic hosts during infection, potentially altering biogeochemical processes mediated by NBT sediment microbes. Our study filled in knowledge gaps on viral communities’ structure, diversity, and metabolic potential in NBT sediments, providing novel insight into viruses’ in hadal habitats.

## Data Availability

The raw metagenomic sequencing data analyzed during the current study are available from the corresponding author on reasonable request.

## Supplementary Materials

The following are available online at https://www.mdpi.com/article/10.3390/genes12070990/s1, Figure S1: An NMDS plot showing variations in the viral community structures of the twelve hadal samples. Figure S2: Venn diagram of shared viral clusters among the five environmental virus data sets (NBT sediment, Tara Ocean seawater, wetland sediment, Stordalen thawing permafrost, and Cold seep). Table S1: Assembly of metagenomic sequencing on the New Britain Trench sediment samples and identification of viral genome/metavirome. Table S2: The relative abundance of virus identified in NBT sediment at a family level. Table S3: Viral community compositions of the NBT sediment and some hadal aquatic habitats. Table S4: Clustered among NBT sediment, cold seep sediments, seawater, wetland, permafrost, and viral RefSeq based on vConTACT2. Table S5: List of eggNOG-mapper annotations for the NBT virome. Table S6: List of putative AMGs of NBT virus.
