# Supplementary material for: Revealing the Viral Community in the Hadal Sediment of the New Britain Trench
Source: Genes (Basel). 2021 Jun 29;12(7):990. doi: 10.3390/genes12070990 (PMC8306916; doi:10.3390/genes12070990)
Supplement: Supplementary file 1 [file genes-12-00990-s001.zip › Supplemental Figure S1 and Supplemental Figure S2.pdf]

genes

MDPI

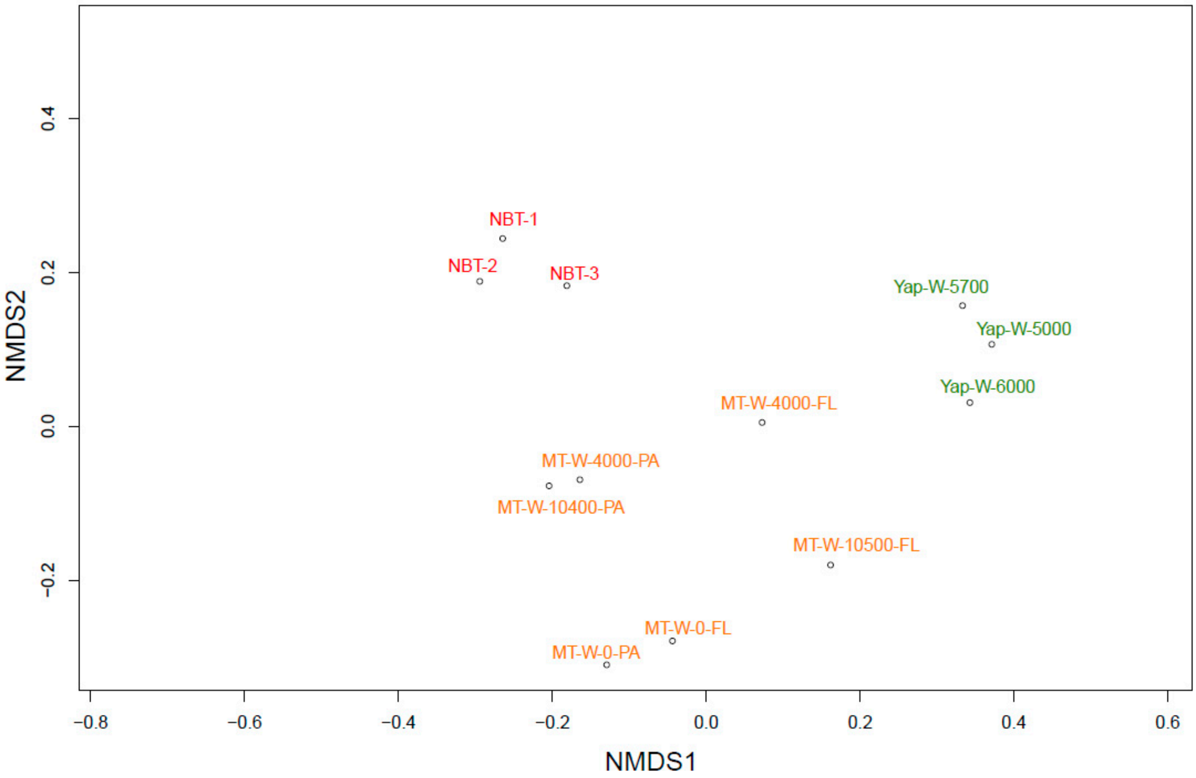

Supplemental Figure S1. An NMDS plot showing variations in the viral community structures of the twelve hadal samples. Sample names are defined by sample location, sample type (seawater is subdivided according size fraction and sediment is subdivided according depth if provided), and sampling depth. MT, Mariana Trench;Yap, Yap Trench; W, seawater; PA ,Particle-attached ; FL, free-living. e.g., MT-W-10500-FL is the free-living fraction of Mariana Trench seawater at 10,500 m, NBT-1, the surficial layer NBT-1 (sediment depth 1-5 cm); NBT-2, the mid layer (sediment depth 5-10 cm); NBT-3, the deep layer (sediment depth 10-15 cm).

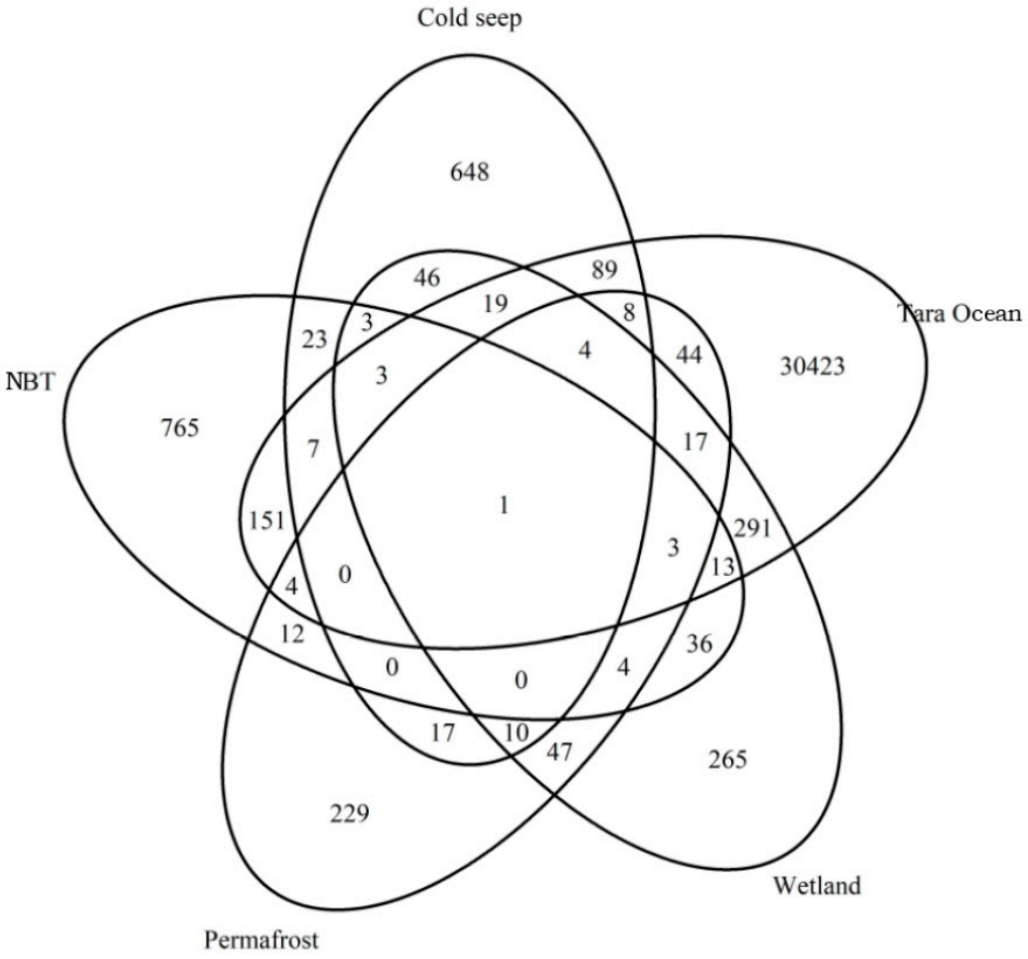

Supplemental Figure S2. Venn diagram of shared viral clusters among the five environmental virus data sets NBT sediment, T ara Ocean seawater, wetland sediment , Stordalen thawing permafrost and Cold seep
